# Clinical results of combined aortic valve-sparing root replacement and mitral valve repair

**DOI:** 10.1093/icvts/ivaf067

**Published:** 2025-03-13

**Authors:** Veronica Lorenz, Jama Jahanyar, Stefano Mastrobuoni, Antonio Segreto, Luca Zanella, Gaby Aphram, Matteo Pettinari, Gebrine El Khoury, Laurent De Kerchove

**Affiliations:** Department of Cardiovascular & Thoracic Surgery, Cliniques Universitaires Saint-Luc, Université Catholique de Louvain, Brussels, Belgium; Department of Cardiovascular & Thoracic Surgery, Cliniques Universitaires Saint-Luc, Université Catholique de Louvain, Brussels, Belgium; Division of Cardiac Surgery, Inova Schar Heart & Vascular Institute, Inova Fairfax Medical Campus, Falls Church, VA, USA; Department of Cardiovascular & Thoracic Surgery, Cliniques Universitaires Saint-Luc, Université Catholique de Louvain, Brussels, Belgium; Department of Cardiovascular & Thoracic Surgery, Cliniques Universitaires Saint-Luc, Université Catholique de Louvain, Brussels, Belgium; U.O.C. Cardiochirurgia, Azienda Ospedaliera Universitaria Policlinico “P. Giaccone”, Palermo, Italy; Department of Cardiovascular & Thoracic Surgery, Cliniques Universitaires Saint-Luc, Université Catholique de Louvain, Brussels, Belgium; Heart Surgery, Department of Medical and Surgical Sciences, University of Bologna, Bologna, Italy; Department of Cardiovascular & Thoracic Surgery, Cliniques Universitaires Saint-Luc, Université Catholique de Louvain, Brussels, Belgium; Department of Cardiovascular & Thoracic Surgery, Cliniques Universitaires Saint-Luc, Université Catholique de Louvain, Brussels, Belgium; Department of Cardiovascular & Thoracic Surgery, Cliniques Universitaires Saint-Luc, Université Catholique de Louvain, Brussels, Belgium; Department of Cardiovascular & Thoracic Surgery, Cliniques Universitaires Saint-Luc, Université Catholique de Louvain, Brussels, Belgium

**Keywords:** aortic surgery, valve sparing, valve repair, connective tissue disease

## Abstract

**OBJECTIVES:**

Aortic valve-sparing root replacement using the reimplantation technique and mitral valve (MV) repair are well-established surgical approaches for the treatment of aortic root pathologies and mitral valve insufficiency. However, the management of concomitant diseases with a dual valve-preserving strategy remains poorly described. Therefore, the aim of this study is to evaluate the long-term outcomes of concomitant valve-sparing surgery and MV repair.

**METHODS:**

This case series includes all the patients who underwent combined valve-sparing root replacement and MV repair at Cliniques Universitaires Saint-Luc (Brussels, Belgium) between January 2000 and June 2022. Actual survival rate and freedom from reoperation were calculated by the Kaplan–Meier method, and the log rank test was used for statistical evaluation.

**RESULTS:**

Forty-five patients were included in the study; they were divided into two groups (13 patients with and 32 patients without connective tissue disorders). There was no hospital mortality. Three patients (7%) required pacemaker implantation. Overall survival at 10 years was 90% (95% confidence interval [CI]: 64–97%). Furthermore, freedom from all reoperations at 10 years was 84% (95% CI: 64–93%). Analysing the two subgroups, we found no statistically significant difference in terms of 10-year survival (log rank *P* = 0.146). However, freedom from reoperation at 10 years was significantly lower in the connective tissue disorder group (63% vs 91%, log rank *P* = 0.031). Most patients treated with transaortic edge-to-edge repair required MV reoperation.

**CONCLUSIONS:**

Combined valve-sparing root replacement with the reimplantation technique and MV operations are complex surgeries. However, they can be performed safely, with excellent long-term survival and repair durability. Applying standard Carpentier techniques for MV repair is crucial, especially in patients with connective tissue disorders.

## GRAPHICAL ABSTRACT

**Figure:**
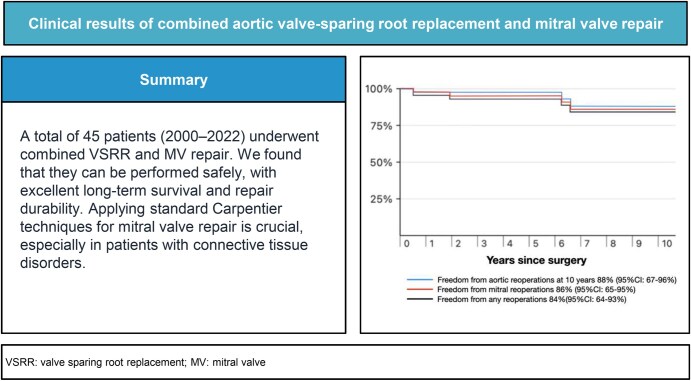

## INTRODUCTION

Aortic valve-sparing root replacement with the reimplantation technique (VSRR) and mitral valve (MV) repair are well-established surgical interventions for the management of aortic root pathologies and mitral insufficiency (MI) [1, 2]. Although these procedures are frequently performed individually, the simultaneous occurrence of both pathologies in a single patient presents a complex clinical challenge. This is particularly evident in patients with connective tissue disorders (CTD), such as Marfan syndrome and Loeys–Dietz syndrome, in which the coexistence of AV and MV abnormalities is more prevalent [3, 4].

While valve-sparing procedures have demonstrated superior outcomes compared to valve replacement—specifically in terms of long-term survival and avoidance of lifelong anticoagulation—there is a paucity of data regarding the combined use of MV repair and VSRR. Current literature on the concomitant performance of these two procedures is limited, with most studies being small-scale [5–9].

This study aims to evaluate our experience with concomitant MV repair and VSRR in a high-volume centre, focusing on the feasibility, safety and clinical outcomes of this combined approach.

## MATERIALS AND METHODS

### Ethical statement

This study was approved by the Ethics Committee of the Cliniques Universitaires Saint-Luc, Brussels, Belgium (2013/03JUI/356). Written informed consent was obtained from all participants. The Ethics Committee approved the establishment for stored data and monitor ongoing use of database.

### Study population

Patients included in this study were selected based on two primary criteria: aortic root dilatation with indication for VSRR [10], who were found to have moderate to severe mitral insufficiency (MI), or patients indicated for mitral repair due to severe MI, who also presented aortic root dilatation during preoperative evaluation.

The underlying mitral pathology was predominantly degenerative, associated with mitral leaflet prolapse, with or without annular dilatation.

Preoperative and operative information were extracted from our prospectively collected institutional clinical and echocardiographic database containing all cardiac procedures performed in our institution.

The primary outcome of the study was survival including in-hospital and late deaths. In-hospital death was defined as any death occurring during the first 30 days after surgery; any other death was considered a late death. Secondary outcomes included AV or MV reoperation. Outcomes were reported for the entire cohort and for different subgroups, according to the presence or absence of connective tissue disorders (CTD vs NCTD).

Operative data are complete, as well as pre- and postoperative characteristics.

Postoperative echocardiograms were performed before discharge from the hospital and annually or every 1–2 years after the first year post-surgery.

Perioperative data were retrospectively reviewed. Clinical and echocardiographic follow-up was collected from hospital and cardiologist reports.

### Operative techniques

All patients underwent surgery via full sternotomy with cardiopulmonary bypass. Warm blood cardioplegia to arrest the heart was given into the aortic root, or directly into the coronary ostia in case of aortic insufficiency (AI). MV was repaired through a standard left atrial approach with posterior leaflet resection techniques, artificial chordae and complete semi-rigid ring annuloplasty. Occasionally, in selected patients with moderate MI and MV disease type 1 or 2 limited to (A2-) P2 segment and to save on operative time, the MV was repaired with an edge-to-edge (Alfieri stitch) performed through the AV. After MV repair, the VSRR was carried out in a standard fashion as previously described by our group using straight tube during early period of the study and Valsalva graft since 2002 (Gelweave and Gelweave Valsalva, Vascutek Ltd, Terumo Company, Renfrewshire, Scotland) [2–15]. There were no differences in outcome when using one or the other prosthesis. Aortic valve lesions, such as cusp prolapse, thickening or large fenestrations were repaired utilizing, respectively, central cusp plication or running suture (Gore-Tex CV-7), shaving and pericardial patch. Occasionally, addition annuloplasty technique (Cabrol Stitch or polytetrafluoroethylene [PTFE] suture annuloplasty) was associated with the reimplantation procedure. PTFE suture annuloplasty (Gore-Tex CV-2, WL Gore and Associates, Flagstaff, AR) was placed around the Dacron graft at the level of the proximal suture line. Suture annuloplasty was tied on the beating heart and under echocardiographic guidance.

All patients were evaluated with a transoesophageal echocardiography to assess the degree of residual AI or MI.

### Statistical analyses

Continuous data are presented as median and interquartile range (IQR). Data for categorical variables are reported as frequency and percentage. Actual survival rate and freedom from reoperation were calculated by the Kaplan–Meier method, and the log rank test was used for statistical evaluation. Patients were censored at the last available follow-up entry. In the survival analysis for recurrence of AI or MI, patients were censored at the last available follow-up echo. Statistical analysis was generated utilizing SPSS (IBM Corporation, Armonk, NY). A *P* value <0.05 was considered statistically significant.

## RESULTS

**Figure 1:**
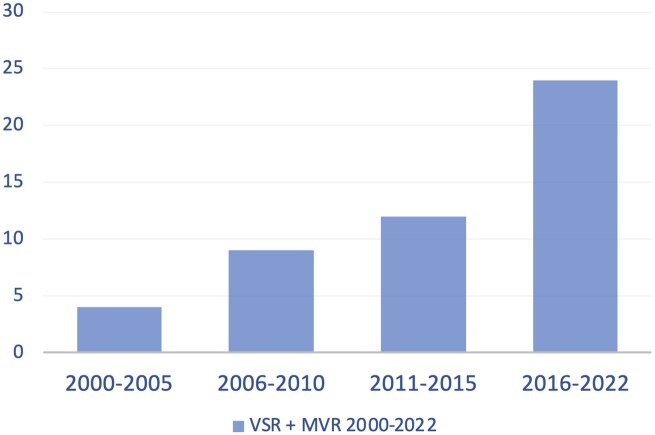
Patients received concomitant VSRR and MV repair between 2000 and 2022.

From January 2000 to June 2022, 652 patients underwent VSRR at the Cliniques Universitaires Saint Luc (Brussels, Belgium). Of these, 45 (7%) patients underwent combined VSRR and MV repair. None of the patients underwent an MV replacement. Patients with other concomitant operations, such as coronary artery bypass grafting (CABG) or tricuspid valve repairs, were also included in the final dataset. Figure 1 shows the rate of interventions based on the year of surgery. Most of the patients (*n* = 32, 71%) were treated in the past decade.

### Patient characteristics

Baseline characteristics and preoperative echocardiographic data are summarized in Table 1.

**Table 1:** Patient characteristics and echocardiographic data

Variables	All (*n* = 45)	NCTD (*n* = 32)	CTD (*n* = 13)	*P* value
Age, years, median [IQR]	55 [35–65]	58 [54–66]	30 [18–39]	<0.001
Male sex, %	40 (89)	30 (94)	10 (77)	0.104
BMI, median [IQR]	24 [22–27]	25 [23–28]	21 [17–23]	<0.001
Previous cardiac surgery	6 (13)	4 (13)	2 (15)	0.796
Hypertension	10 (22)	8 (25)	2 (15)	0.482
Renal failure (creat >1.3 mg/dl)	5 (11)	5 (16)	0	0.131
Diabetes mellitus	0	0	0	
Hyperlipidaemia	7 (16)	6 (19)	1 (8)	0.354
COPD	4 (9)	3 (9)	1 (8)	0.857
NYHA				
I–II	37 (82)	27 (84)	10 (77)	0.553
III–IV	8 (18)	5 (16)	3 (23)	0.553
Aortic insufficiency				
1	20 (44)	11 (34)	9 (69)	0.033
2	9 (20)	5 (16)	4 (31)	0.250
≥3	16 (36)	16 (50)	0	0.001
Bicuspid aortic valve	5 (11)	5 (16)	0	0.131
Aortic root diameter, median [IQR]	50 [47–54]	51 [47–54]	49 [47–57]	0.486
Left ventricular ejection fraction				
≥50%	36 (80)	26 (81)	10 (77)	0.742
31–50%	9 (20)	6 (19)	3 (23)	0.742
Mitral insufficiency				
Moderate	7 (16)	3 (9)	4 (31)	0.073
Severe	38 (84)	29 (91)	9 (69)	0.073
Connective tissue disease				
Marfan			11 (24)	
Loeys–Dietz			1 (2)	
Ehlers–Danlos			6 (13)	

Percentages are shown in parentheses unless indicated as IQR.

Abbreviation: COPD = chronic obstructive pulmonary disease. NYHA = New York Heart Association.

Forty (89%) patients were male, and median age was 55 (IQR: 35–65) years. Thirteen patients (29%) had a genetic diagnosis of CTD, of which the majority (*n* = 11) had Marfan syndrome (one Loeys–Dietz and one Ehlers-Danlos syndrome). In addition, six patients had previously undergone cardiac surgery.

The median aortic root diameter was 50 (47–54) mm. The aortic valve (AV) was bicuspid in five patients (11%). Precisely, 36% of patients had an associated AI greater than moderate. Severe MI was found in 86% of patients (14% had moderate MI).

As shown in Table 2, all patients underwent aortic root valve-sparing surgery with the reimplantation technique (David procedure). Valsalva graft was used in majority of the patients (*n* = 41, 91%). Twenty-seven patients (60%) needed aortic leaflet repair. Cabrol annuloplasty stitches were applied in three (7%) patients and PTFE suture annuloplasty in two patients (4%).

**Table 2:** Operative data

Variables	All (*n* = 45)	NCTD (*n* = 32)	CTD (*n* = 13)	*P* value
Aortic repair				
Valve-sparing reimplantation	45 (100)	32 (100)	13 (100)	
** **Straight tube	4 (9)	4 (13)	0	0.182
** **Valsalva tube (Gelweave or Cardioroot)	41 (91)	28 (88)	13 (100)	0.182
Aortic leaflet repair	27 (60)	21 (66)	6 (46)	0.227
** **Central plication	16 (36)	14 (44)	2 (15)	0.072
** **Running suture	7 (16)	5 (16)	2 (15)	0.984
** **Shaving	8 (18)	6 (19)	2 (15)	0.789
** **Patch reconstruction	1 (2)	1 (3)	0	0.519
Mitral repair				
** **Annuloplasty ring only	12 (27)	10 (31)	2 (15)	0.275
** **Neochordae + annuloplasty ring	3 (7)	3 (9)	0	0.253
** **Leaflet resection + annuloplasty ring	17 (38)	10 (31)	7 (54)	0.156
** **Leaflet resection + neochordae + annuloplasty ring	4 (9)	3 (9)	1 (8)	0.857
** **Leaflet resection no annuloplasty	2 (4)	2 (6)	0	0.356
** **Commissural edge-to-edge (no ring)	2 (4)	2 (6)	0	0.356
** **Trans-aortic edge-to-edge	5 (11)	2 (6)	3 (23)	0.104
Concomitant procedures	11 (24)	10 (31)	1 (8)	0.096
** **Tricuspid valve repair	2 (4)	2 (6)	0	0.356
** **Maze procedure	4 (9)	4 (13)	0	0.182
** **CABG	2 (4)	2 (6)	0	0.356
** **Hemiarch replacement	1 (2)	1 (3)	0	0.519
Cross-clamp time, median [IQR] min	145 [131–165]	145 [138–165]	134 [118–154]	0.089
Cardiopulmonary bypass time, median [IQR] min	170 [151–202]	176 [156–203]	170 [137–193]	0.444

Percentages are shown in parentheses unless indicated as IQR.

Regarding the MV repair, a complete annuloplasty ring was used in 36 patients (80%). Neochords were used in 7 (16%) patients, and posterior leaflet resection techniques were performed in 23 (51%) patients. Transaortic edge-to-edge MV repair was performed in five (11%) patients, whose diagnosis of moderate MI was made in the operating room before the operation.

Other additional procedures and operative parameters are summarized in Table 2.

### Early postoperative outcomes

There was no hospital mortality during index procedure. Postoperative complications are presented in Table 3. One patient had a stroke, and three (7%) patients underwent permanent pacemaker implantation. Precisely, 98% of patients were discharged with a trivial-mild mitral and AI after an average of 8 days of hospital stay.

**Table 3:** 30-days outcomes

Variables	All (*n* = 45)	NCTD (*n* = 32)	CTD (*n* = 13)	*P* value
Mortality	0	0	0	
Postoperative new AF	8 (18)	7 (22)	1 (8)	0.259
Pacemaker implantation	3 (7)	2 (6)	1 (8)	0.860
Stroke^a^	1 (2)	1 (3)	0	0.519
Hospital stay (days), median [IQR]	11 [8–15]	11 [8–15]	13 [11–13]	0.350
Discharge echo				
Aortic insufficiency				
** **0–1	44 (98)	31 (97)	13 (100)	0.519
** **2	1 (2)	1 (3)	0	0.519
** **≥3	0	0	0	
Mitral insufficiency				
** **Trivial-mild	44 (98)	32 (100)	12 (92)	0.113
** **Moderate	1 (2)	0	1 (8)	0.113
** **Severe	0	0	0	

a Intraoperative aortic dissection during aortic cannulation causing disabling stroke on right carotid dissection and hemiplegia.

Abbreviation: AF = atrial fibrillation.

### Late outcomes

**Figure 2:**
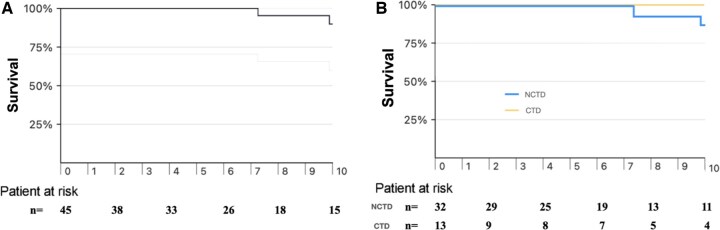
(**A**) Survival following concomitant VSR and MV repair. Estimated survival at 10 years was 89.6% (95% CI: 64.3–97.3%). (**B**) Survival following concomitant VSRR and MVR in NCTD vs CTD. Estimated survival at 10 years was 86% (95% CI: 54–96%) for NCTD and 100% for CTD (log rank *P* = 0.393).

The median follow-up duration was 6.5 years, and 15 patients were still at risk at 10 years. A total of five deaths occurred during the follow-up period, three after 10 years (at 11, 14 and 16 years, respectively). One patient died from mitral endocarditis 14 years after surgery; another patient died from unknown cause (by the age of 85), and the remaining three patients died from non-cardiac causes (one pneumonia, one Covid infection, one car accident). Overall survival at 10 years was 90% (95% confidence interval [CI]: 64–97%) (Fig. 2A). Analysing the two subgroups, the 10 years survival was 100% for CTD and 86% (95% CI: 54–96%) for NCTD, with no statistically significant difference (log rank *P* = 0.393) (Fig. 2B).

During the follow-up period, seven patients required reintervention: two patients on the AV, three on the MV and two patients had reoperation on both AV and MV (Table 4). The linearized risk of reintervention was 2.1%.

**Table 4:** Follow-up outcomes

Outcome	All (*n* = 44)	NCTD (*n* = 31)	CTD (*n* = 13)
Late death	5 (11)	5 (16)	0
Reoperations	7 (16)	3 (10)	4 (31)
** **Aortic alone	2 (5)	1 (3)^a^	1 (8)^b^
** **Mitral alone	3 (7)	1 (3)^c^	2 (15)^d^
** **Aortic + mitral	2 (5)	1 (3)^b^,d	1 (8)^e^
Last echocardiography	*N* = 34	NCTD = 27	CTD = 7
** **Aortic insufficiency			
** **0–I	31 (91)	24 (89)	7 (100)
** **II	3 (9)	3 (11)	0
** **III–IV	0	0	0
** **Mitral insufficiency			
** **Trivial-mild	28 (82)	22 (82)	6 (86)
** **Moderate	5 (15)	5 (19)	0
** ** Severe	1 (3)	0	1 (14)^d^

a Aortic stenosis 13 years after BAV repair.

b Recurrent AI, after initial AV repair with running suture on the three cusps.

c Recurrent MI after initial MV repair by commissural edge-to-edge without annuloplasty ring.

d Recurrent MI after initial MV repair with transaortic edge-to-edge.

e Recurrent MI after initial MV repair with P2 resection and annuloplasty ring and recurrent AI after AV repair with running suture for right coronary cusp prolapse.

Regarding the four patients needing AV reintervention, one was reoperated for stenosis 13 years after bicuspid aortic valve (BAV) repair and the three others for AI. Two of them had had three cusps repaired with running suture, and the last one had right coronary cusp prolapse treated with a running suture (Table 4). In recent years, we have avoided the use of free-edge running sutures because we found to be a risk factor for stenosis and failure of aortic repair [16]. Aortic valve re-repair was performed in one patient, while the remaining three received AV replacement.

Regarding the patients needing MV reintervention, Table 4 shows the causes of recurrence of MI. All five patients were reoperated for severe recurrent MI. Three of them had as initial MV repair technique a transaortic edge-to-edge, one had a commissural edge-to-edge without annuloplasty ring and one had a P2 resection plus annuloplasty ring. At reoperation, all patients underwent successful re-repair with posterior leaflet resection or artificial chordae and annuloplasty ring. All seven patients survived their reoperation.

**Figure 3:**
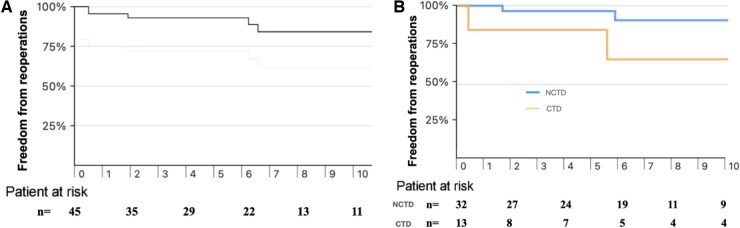
(**A**) Freedom from reoperation following concomitant VSR and MV repair at 10 years was 84% (95% CI: 64–93%). (**B**) Freedom from reoperation after VSRR and MVR at 10 years was 91% (95% CI: 66–98%) for NCTD and 63% (95% CI: 17–88%) for CTD (log rank *P* = 0.031).

Freedom from AV and MV reoperations at 10 years for the entire cohort was 84% (95% CI: 64–93%) (Fig. 3A). At 10 years, freedom from reoperation was significantly lower in the CTD group (63% [95% CI: 17–88%]) compared to the NCTD group (91% [95% CI: 66–98%]) (log rank *P* = 0.031) (Fig. 3B).

Competing risk curves are reported in the Supplementary Material (Supplementary Fig. S1).

On last echocardiographic follow-up, one patient (transaortic edge-to-edge MV repair) had developed severe MI and has not been reoperated yet by the patient’s decision. In the whole cohort, also considering the patients who underwent reintervention, 10 years freedom from moderate to severe AI was 90% (95% CI: 70–97%) and freedom from moderate to severe MI was 86% (95% CI: 68–95%) (Kaplan–Meier curves are reported as Supplementary Figs S2 and S3).

During follow-up, one patient had a thromboembolic event, another had endocarditis. Linearized rates were 0.3% for both events. Major bleeding occurred in two patients with a linearized rate of 0.6%.

## DISCUSSION

This study evaluates the early and long-term outcomes of patients who underwent concomitant VSRR and MV repair. Valve-preserving operations, especially of the AV, are technically demanding and less frequently performed compared to traditional AV replacement. This is largely due to the intricate nature of maintaining a functional AV while addressing root dilation, as well as concerns about potential postoperative valve dysfunction. In a similar fashion, the decision to also repair the MV adds complexity and requires specialized techniques, although nowadays it is the gold standard at many cardiac surgery centres worldwide [2, 17, 18].

Historically, replacement of the aortic root with a composite valve graft utilizing a mechanical prosthesis has been the go-to procedure for young patients, with or without CTD [19–21]. These facts explain that few studies to date have reported the outcome of large series of combined VSRR and MV repair. The results we show in this nearly unique cohort of patients are favourably compared to literature data regarding several outcomes. Specifically, we report 100% survival at 1 and 5 years, and 90% at 10 years, which is comparable to prior studies that focused on double valve preservation. In a study by David *et al.* [7], 123 patients underwent combined aortic root replacement with MV surgery. Although in this cohort the AV was only preserved in 21 patients and MV repair was performed in 67 patients, the authors demonstrated that the lowest mortality rate occurred in patients with a first-time VSRR combined with MV repair.

Nicolini *et al.* published a series of 1167 double AV and MV replacements in 2014. Of these, 443 underwent double mechanical valve replacements and hospital mortality for patients younger than 70 years was 4.8% and 1-year mortality was 7.6%. Interestingly, survival for the entire cohort at 5 years was only 75.1% [22].

In another study, Coutinho *et al.* [23], reported a 6-year survival of 76.7% after aortic and mitral replacement (45.6% of the cohort had a double mechanical valve).

Finally, in another study by Caus *et al.* [24], the authors analysed long-term follow-up in a matched population of patients treated with double biological or mechanical prosthesis. For the mechanical group, the results were statistically better compared to biological valves. The mean age was 60-year-old, and survival was 78.9% and 62.3% at 5- and 10 years, respectively. The 10-year freedom from reoperation was 80.6%. The cumulative incidence of thromboembolic and haemorrhagic events was 8% at 5 years, 23.6% at 10 years and 70.6% at 15 years. Overall, disappointing results with prosthetic double valve replacements. This explains that, in recent decades, VSRR has become increasingly popular and affords us the ability to avoid prosthetic AVs with its prosthesis-related complications and its need for chronic anticoagulation [13, 25–28].

Herein, we are sharing one of the largest experiences reported to date with combined VSRR and MV repair including almost 30% of the patients with CTD. The Stanford group has previously reported that 15–20% of patients with aortic root dilation in Marfan patients also presented with significant MV disease requiring surgical correction [29].

In patients with CTD, we found no mortality up to 10 years, likely attributable to the younger age of this cohort.

However, the reoperation rate in this cohort was substantial, with 16% requiring a reoperation at 10 years. This was particularly notable in the CTD group, where the incidence of reoperation was higher, likely due to the progressive nature of the underlying disease [30]. These findings are in line with prior studies on double valve replacements, where reoperation rates increase over time, particularly in patients who underwent mechanical valve replacement [22–24].

Analysing reintervention in detail, our study suggests that the long-term outcomes of combined VSRR and MV repair can be optimized by employing more durable mitral repair techniques. In our study, most patients who failed MV repair and required reoperation underwent initial repair without ring or with the edge-to-edge technique. This highlights the notion that, for each patient, a repair of the MV following the basic principles set forth by Alan Carpentier is crucial. Although sometimes the pathology allows to approach the MV repair through the AV with a simple edge-to-edge repair, we have learned that this approach is not as durable in this patient population and leads to failure early on. Hence, performing a suboptimal repair to limit cardiopulmonary bypass time comes with its own price. Therefore, we now approach these patients with standard MV repair with a complete or incomplete semi-rigid ring. The more complex repairs did not add to mortality or postoperative complications, and our freedom from moderate MI rates at mid-term follow-up was totally acceptable. Importantly, every patient who had an MV valve reoperation underwent a successful re-repair using standard Carpentier techniques.

Regarding AV sparing procedures, more than 50% of the patients in this cohort also required leaflets repairs. During reoperation for recurrent AI, we noted that three patients had undergone one or multiple leaflet prolapse repair with running suture. This technique, however, is rarely used today; leaflet prolapse being principally repaired with central plication.

Interestingly, the fourth patient in this cohort developed aortic stenosis one decade after VSRR, which is consistent with the known long-term progression of AV disease, especially in the context of BAV pathology [16, 31].

Recent interest has been increased in alternative techniques such as the personalized external aortic root support (PEARS), for the management of moderate root aneurysm [32–35]. In 2016, Benedetto *et al.* [34] described the concomitant use of PEARS and MV repair in two patients. In a multicentre cohort study evaluating 200 consecutive patients treated with PEARS, the most frequent concomitant procedure was mitral repair (10%) [32]. The follow-up is still short, and the authors do not provide information about mitral function during follow-up. Compared to PEARS, we believe that VSRR is a more versatile intervention, because it allows for the repair of the aortic leaflets as well what is often needed especially in case of significant preoperative AI [36].

Finally, we have to mention that, in patients with CTD, the reimplantation technique is also more durable than the remodelling technique [13, 37], which has also been supported by the Toronto group [38]. The remodelling technique leaves areas of weakness in the functional aortic annulus, which are prone to disease recurrence. These areas however, become functionally excluded with the Reimplantation technique.

### Study limitations

Although data were gathered in a prospective manner, the retrospective nature of the analysis introduces some limitations, including the potential for selection bias and confounding factors, which may affect the strength of causal conclusions. Moreover, this study is limited by the relatively small sample size and the long study period, which covers 20 years, though most patients were treated in the past decade. The median follow-up period was relatively short, and both follow-up and the last echocardiographic follow-up were not standardized in terms of timing, which could impact consistency in outcome assessment. Additionally, the two study groups were analysed primarily for descriptive purposes due to the limited number of patients. Finally, since all surgeries were performed at a single centre by a limited number of highly experienced surgeons, the results may not be easily generalizable to all institutions or surgical teams.

## CONCLUSIONS

In conclusion, our study supports the safety and effectiveness of concomitant VSRR and MV repair. However, reoperation remains a concern, especially in patients with connective tissue disorders. The use of classic Carpentier repair techniques with annular stabilization is crucial, while methods like the edge-to-edge repair should be avoided, as they may offer short-term competence but jeopardize long-term valve durability.

## Supplementary Material

ivaf067_Supplementary_Data

## Data Availability

The data underlying this article will be shared on reasonable request to the corresponding author.

## Notes

Presented at the HVS Annual Meeting, Boston, February 18–21, 2024.

## ABBREVIATIONS

AI: Aortic insufficiency
AV: Aortic valve
CI: Confidence interval
CTD: Connective tissue disorders
IQR: Interquartile range
MI: Mitral insufficiency
MV: Mitral valve
NCTD: Non-connective tissue disorders
PEARS: Personalized external aortic root support
VSRR: Aortic valve-sparing root replacement with the reimplantation technique

## References

[1] Mastrobuoni S , de Kerchove L, Navarra E et al Long-term experience with valve-sparing reimplantation technique for the treatment of aortic aneurysm and aortic regurgitation. J Thorac Cardiovasc Surg 2019;158:14–23. 30635185 10.1016/j.jtcvs.2018.10.155

[2] David TE , David CM, Tsang W, Lafreniere-Roula M, Manlhiot C. Long-term results of mitral valve repair for regurgitation due to leaflet prolapse. J Am Coll Cardiol 2019;74:1044–53. 31439213 10.1016/j.jacc.2019.06.052

[3] Koda Y , Kawamoto T, Yokawa K et al Mid-term outcomes of simultaneous mitral valve repair in patients with miral regurgitation and concomitant annulo-aortic ectasia. Gen Thorac Cardiovasc Surg 2019;67:1014–20. 31041727 10.1007/s11748-019-01129-z

[4] Kunkala MR , Schaff HV, Li Z et al Mitral valve disease in patients with Marfan syndrome undergoing aortic root replacement.Circulation 2013;128:S243–7 24030414 10.1161/CIRCULATIONAHA.112.000113

[5] Imielski BR , Soletti G, Rahouma M et al The impact of concomitant mitral valve intervention on outcomes of aortic root replacement. J Card Surg 2022;37:4662–9. 36273410 10.1111/jocs.17056

[6] Javadikasgari H , Roselli EE, Aftab M et al Combined aortic root replacement and mitral valve surgery: the quest to preserve both valves. J Thorac Cardiovasc Surg 2017;153:1023–30.e1. 28262292 10.1016/j.jtcvs.2017.01.006

[7] David TE , Armstrong S, Maganti M, Ihlberg L. Clinical outcomes of combined aortic root replacement with mitral valve surgery. J Thorac Cardiovasc Surg 2008;136:82–7. 18603058 10.1016/j.jtcvs.2008.02.038

[8] Beyersdorf F. Special considerations in mitral valve repair during aortic root surgery. Ann Cardiothorac Surg 2015;4:487–91. 26539359 10.3978/j.issn.2225-319X.2014.12.08 PMC4598466

[9] David TE , Armstrong S, Manlhiot C, McCrindle BW, Feindel CM. Long-term results of aortic root repair using the reimplantation technique. J Thorac Cardiovasc Surg 2013;145:S22–5 23260437 10.1016/j.jtcvs.2012.11.075

[10] Czerny M , Grabenwöger M, Berger T et al; EACTS/STS Scientific Document Group. EACTS/STS guidelines for diagnosing and treating acute and chronic syndromes of the aortic organ. Eur J Cardiothorac Surg 2024;65:ezad426. 38408364 10.1093/ejcts/ezad426

[11] Aphram G , Tamer S, Mastrobuoni S, El Khoury G, de Kerchove L. Valve sparing root replacement: reimplantation of the aortic valve. Ann Cardiothorac Surg 2019;8:415–7. 31240189 10.21037/acs.2019.04.05 PMC6562083

[12] El Khoury G , de Kerchove L. Principles of aortic valve repair. J Thorac Cardiovasc Surg 2013;145:S26–9. 23260436 10.1016/j.jtcvs.2012.11.071

[13] Jahanyar J , de Kerchove L, Arabkhani B et al Three decades of reimplantation of the aortic valve-the Brussels experience. Ann Cardiothorac Surg 2023;12:244–52. 37304697 10.21037/acs-2023-avs1-23 PMC10248911

[14] Jahanyar J , El Khoury G, de Kerchove L. Reimplantation should be the gold standard to treat the regurgitant bicuspid aortic valve. JTCVS Tech 2022;13:42–3. 35711183 10.1016/j.xjtc.2022.02.038 PMC9196934

[15] Jahanyar J , de Kerchove L, El Khoury G. Bicuspid aortic valve repair: the 180°-reimplantation technique. Ann Cardiothorac Surg 2022;11:473–81. 35958541 10.21037/acs-2022-bav-18 PMC9357964

[16] Spadaccio C , Nenna A, Henkens A et al Predictors of long-term stenosis in bicuspid aortic valve repair. J Thorac Cardiovasc Surg 2024;167:611–21.e6. 35659121 10.1016/j.jtcvs.2022.04.024

[17] David TE , Park J, Steve Fan CP. Mitral valve surgery in patients with Marfan syndrome. J Thorac Cardiovasc Surg 2024;169:599–605. 38678476 10.1016/j.jtcvs.2024.01.046

[18] Castillo JG , Anyanwu AC, Fuster V, Adams DH. A near 100% repair rate for mitral valve prolapse is achievable in a reference center: implications for future guidelines. J Thorac Cardiovasc Surg 2012;144:308–12. 22698565 10.1016/j.jtcvs.2011.12.054

[19] Coselli JS , Volguina IV, Nguyen L, Green SY, LeMaire SA, Moon MR. Outcomes of aortic root replacement in patients with Marfan syndrome: the role of valve-sparing and valve-replacing approaches. Ann Cardiothorac Surg 2023;12:338–49. 37554715 10.21037/acs-2023-avs2-0085 PMC10405346

[20] Goldstone AB , Chiu P, Baiocchi M et al Mechanical or biologic prostheses for aortic-valve and mitral-valve replacement. N Engl J Med 2017;377:1847–57. 29117490 10.1056/NEJMoa1613792 PMC9856242

[21] Gerdisch MW , Sathyamoorthy M, Michelena HI. The role of mechanical valves in the aortic position in the era of bioprostheses and TAVR: evidence-based appraisal and focus on the On-X valve. Prog Cardiovasc Dis 2022;72:31–40. 35738422 10.1016/j.pcad.2022.06.001

[22] Nicolini F , Agostinelli A, Fortuna D et al Outcomes of patients undergoing concomitant mitral and aortic valve surgery: results from an Italian regional cardiac surgery registry. Interact CardioVasc Thorac Surg 2014;19:763–70. 25082836 10.1093/icvts/ivu248

[23] Coutinho GF , Martínez Cereijo JM, Correia PM et al Long-term results after concomitant mitral and aortic valve surgery: repair or replacement? Eur J Cardiothorac Surg 2018;54:1085–92. 29800093 10.1093/ejcts/ezy205

[24] Caus T , Rouvière P, Collart F, Mouly-Bandini A, Montiès JR, Mesana T. Late results of double-valve replacement with biologic or mechanical prostheses. Ann Thorac Surg 2001;71:S261–4. 11388200 10.1016/s0003-4975(01)02499-7

[25] Jahanyar J , de Kerchove L, Tsai PI et al Patient selection for aortic valve-sparing operations. Ann Cardiothorac Surg 2023;12:259–61. 37304694 10.21037/acs-2023-avs1-19 PMC10248909

[26] Jahanyar J , Tsai PI, Arabkhani B et al Functional and pathomorphological anatomy of the aortic valve and root for aortic valve sparing surgery in tricuspid and bicuspid aortic valves. Ann Cardiothorac Surg 2023;12:179–93. 37304696 10.21037/acs-2023-avs1-22 PMC10248914

[27] Otto CM , Nishimura RA, Bonow RO et al 2020 ACC/AHA guideline for the management of patients with valvular heart disease: executive summary: a report of the American College of Cardiology/American Heart Association Joint Committee on Clinical Practice Guidelines. Circulation 2021;143:e35–e7. 33332149 10.1161/CIR.0000000000000932

[28] Tirone D , Carolyn D, Feindel CM, Manlhiot C. Reimplantation of the aortic valve at 20 years. J Thorac Cardiovasc Surg 2017;153:232–8. 27923487 10.1016/j.jtcvs.2016.10.081

[29] Demers P , Liang D, Miller DC. Images in cardiovascular medicine. Simultaneous "Tirone David-V" valve-sparing aortic root replacement and radical mitral valve repair for the Marfan syndrome with Barlow syndrome. Circulation 2003;108:e116–7. 14568889 10.1161/01.CIR.0000092236.67713.04

[30] Lawrence KM , Bavaria JE, Kelly JJ et al Mitral valve repair is effective when performed with valve-sparing aortic root replacement. Ann Thorac Surg Short Rep 2023;1:638–41. 39790660 10.1016/j.atssr.2023.06.008 PMC11708686

[31] Schneider U , Feldner SK, Hofmann C et al Two decades of experience with root remodeling and valve repair for bicuspid aortic valves. J Thorac Cardiovasc Surg 2017;153:S65–71. 28168982 10.1016/j.jtcvs.2016.12.030

[32] Van Hoof L , Rega F, Golesworthy T et al Personalised external aortic root support for elective treatment of aortic root dilation in 200 patients. Heart 2021;107:1790–5. 34326135 10.1136/heartjnl-2021-319300

[33] Treasure T , Petrou M, Rosendahl U et al Personalized external aortic root support: a review of the current status. Eur J Cardiothorac Surg 2016;50:400–4. 27032474 10.1093/ejcts/ezw078

[34] Benedetto U , Jin XY, Hill E, Treasure T, Petrou M. An option for concomitant management of moderate Marfan root aneurysm at the time of mitral valve repair: a role for personalized external aortic root support. Ann Thorac Surg 2016;102:e499–e501. 27847065 10.1016/j.athoracsur.2016.05.031

[35] Nemec P , Pepper J, Fila P. Personalized external aortic root support. Interact CardioVasc Thorac Surg 2020;31:342–5. 32761056 10.1093/icvts/ivaa111

[36] Valo J , Jokinen JJ, Kaarne M, Ihlberg L. Expanding indications for valve-sparing aortic root reconstruction: early and midterm results. Ann Thorac Surg 2013;95:579–85. 23103004 10.1016/j.athoracsur.2012.08.079

[37] Jahanyar J , de Kerchove L, Munoz DE, El Khoury G. Twenty-year follow-up after valve-sparing aortic root replacement with the Yacoub or David procedure in Marfan patients. JTCVS Open 2021;7:47–9. 36003717 10.1016/j.xjon.2021.07.013 PMC9390185

[38] Elbatarny M , David TE, David CM, Chung JCY, Lafreniere-Roula M, Ouzounian M. Improved outcomes of reimplantation vs remodeling in marfan syndrome: a propensity-matched study. Ann Thorac Surg 2023;115:576–82. 35841950 10.1016/j.athoracsur.2022.05.068

