# Supplementary material for: Clinical results of combined aortic valve-sparing root replacement and mitral valve repair
Source: Interdiscip Cardiovasc Thorac Surg. 2025 Mar 13;40(4):ivaf067. doi: 10.1093/icvts/ivaf067 (PMC11955238; doi:10.1093/icvts/ivaf067)
Supplement: ivaf067_Supplementary_Data [file ivaf067_supplementary_data.zip › supplemental material.docx]

**Supplemental material**:

Supplemental material Figure 1: competing risk curve. 1: death, 2: reoperation.

Supplemental material Figure 2: Freedom from Moderate-to-Severe Aortic Insufficiency

Supplemental material Figure 3: Freedom from Moderate-to-Severe Mitral Insufficiency
