# Supplementary figures and images for: Clinical results of combined aortic valve-sparing root replacement and mitral valve repair
Source: Interdiscip Cardiovasc Thorac Surg. 2025 Mar 13;40(4):ivaf067. doi: 10.1093/icvts/ivaf067 (PMC11955238; doi:10.1093/icvts/ivaf067)

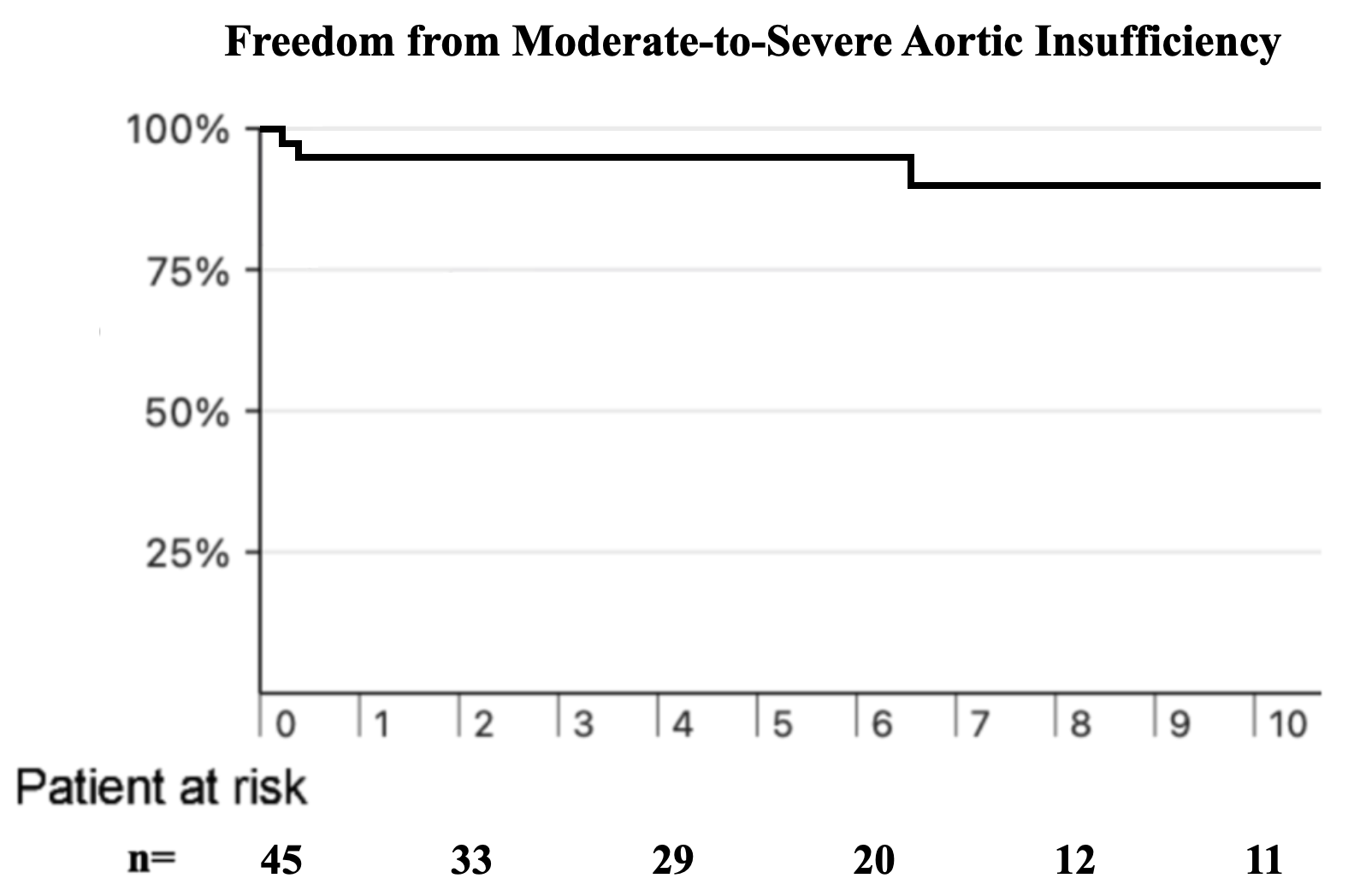

Supplement: ivaf067_Supplementary_Data [file ivaf067_supplementary_data.zip › Supplemental Material Figure 2.png]

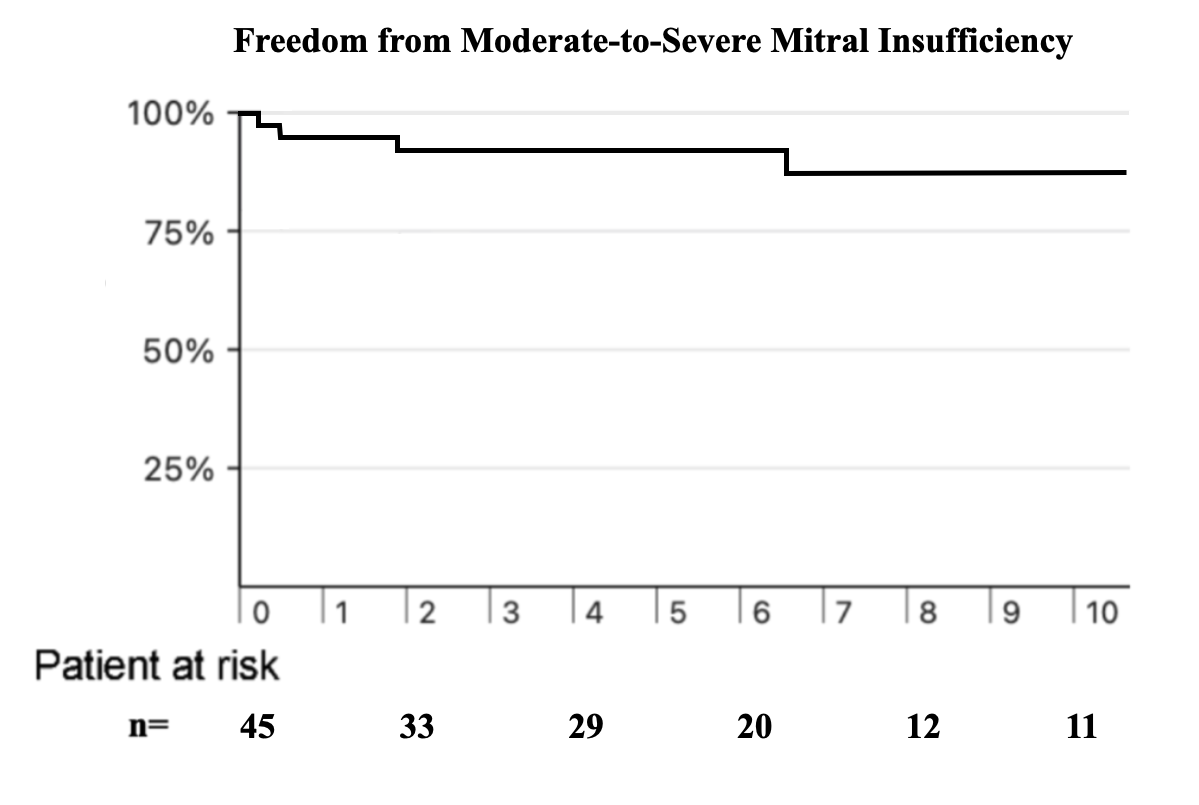

Supplement: ivaf067_Supplementary_Data [file ivaf067_supplementary_data.zip › Supplemental Material Figure 3.png]
